# Associations between depression and the incident risk of obesity in southwest China: A community population prospective cohort study

**DOI:** 10.3389/fpubh.2023.1103953

**Published:** 2023-01-19

**Authors:** Tao Liu, Bo Wu, Yuntong Yao, Yun Chen, Jie Zhou, Kelin Xu, Na Wang, Chaowei Fu

**Affiliations:** ^1^Guizhou Center for Disease Control and Prevention, Guiyang, China; ^2^School of Public Health, Fudan University, Shanghai, China; ^3^National Health Commission of People's Republic of China (NHC) Key Laboratory of Health Technology Assessment, Fudan University, Shanghai, China

**Keywords:** depression, obesity, prospective cohort study, community population, Chinese

## Abstract

**Objective:**

This study aimed to describe the incidence of obesity and investigate associations between depression and the risk of incident obesity among residents in Southwest China.

**Methods:**

A 10-year prospective cohort study of 4,745 non-obese adults was conducted in Guizhou, southwest China from 2010 to 2020. Depression was assessed by the Patient Health Questionnaire-9 (PHQ-9) while the obesity was identified by waist circumference (WC) and/or body mass index (BMI). Cox proportional hazard models were used to estimate hazard ratios (HR), and 95% confidence intervals (CIs) of depression and incident obesity.

**Results:**

A total of 1,115 incident obesity were identified over an average follow-up of 7.19 years, with an incidence of 32.66 per 1,000 PYs for any obesity, 31.14 per 1,000 PYs and 9.40 per 1,000 PYs for abdominal obesity and general obesity, respectively. After adjustment for potential confounding factors, risks of incident abdominal obesity for subjects with minimal (aHR: 1.22, 95% CI: 1.05, 1.43), and mild or more advanced depression (aHR: 1.27, 95% CI: 1.01, 1.62) were statistically higher than those not depressed, while there was no significant association with incident general obesity. The risks of any incident obesity among subjects with minimal (aHR: 1.21, 95% CI: 1.04, 1.40), mild or more advanced depression (aHR: 1.30, 95% CI: 1.03, 1.64) were significantly higher than those not depressed and positive association was found for PHQ score per SD increase (aHR: 1.07, 95%CI: 1.01, 1.13), too. The association was stronger significantly in Han Chinese (minimal: aHR: 1.27, 95% CI: 1.05, 1.52; mild or more advanced: aHR: 1.70, 95% CI: 1.30, 2.21) and farmers (minimal: aHR: 1.64, 95% CI: 1.35, 2.01; mild or more advanced: aHR: 1.82, 95% CI: 1.32, 2.51).

**Conclusion:**

Depression increased the risk of incident obesity among adults in Southwest China, especially among Han Chinese and farmers. This finding suggests that preventing and controlling depression may benefit the control of incident obesity.

## 1. Introduction

Obesity is one of the critical public health challenges worldwide. With socioeconomic growth and lifestyle changes, World Health Organization reported that the global age-standardized prevalence of overweight and obesity was high as 39 and 13% among adults in 2016 (1). As a significant risk factor for multiple chronic diseases such as diabetes, metabolic syndrome, musculoskeletal disorders, and some cancers, obesity caused 4 million deaths and 120 million disability-adjusted life years worldwide in 2015 (2, 3). However, extensive documentation indicated that the distribution of body mass index (BMI) and average waist circumference (WC) have shifted upward (4). Obesity incidence in America had increased more than 3-fold from 5.8 to 14.8% during 1950–2000 (5), while similar trends emerged in numerous low-income and middle-income countries (6). In China, the prevalence of overweight and obesity was 34.3 and 16.4%, which increased by 13.9 and 37.8% from 2012 to 2020 (7).

Overweight and obesity are influenced by socioeconomic status, diet, and environment (2, 8). Mental disorders were also frequently mentioned in recent studies (9). Several epidemiological studies have confirmed the complex mechanism of depression-to-obesity pathways, but the evidence was mixed and varied across regions or races. The role of gender was equivocal in the association between depression and obesity, which varied among Chinese adolescents, middle-aged residents, and American (10–12). Some studies found that non-Latino and white conferred a higher risk of comorbid obesity and mood disorders compared to Latino, African–American, and Asian Socio-cultural factors in different areas may also affect the relationship between obesity and depression (11–13). A meta-analysis found depressed persons had a 58% increased risk of becoming obese (14), while another Dutch study showed that the presence of baseline depressive symptoms was not prospectively associated with the development of obesity (15). Most studies on the association between depression and incident obesity were cross-sectional studies whose findings varied over age in China (10, 12, 13, 16, 17), which could not make the causal association between depression status and obesity.

To our knowledge, prospective cohort studies covering adults to evaluate the risk of incident obesity based on different depressive states have not been reported in China. Based on Guizhou Population Health Cohort (18), this study aimed to explore the association between depression and incident obesity by analyzing the discrepancies in obesity outcomes among people with different depression levels.

## 2. Materials and methods

### 2.1. Study design and population

**Figure 1:**
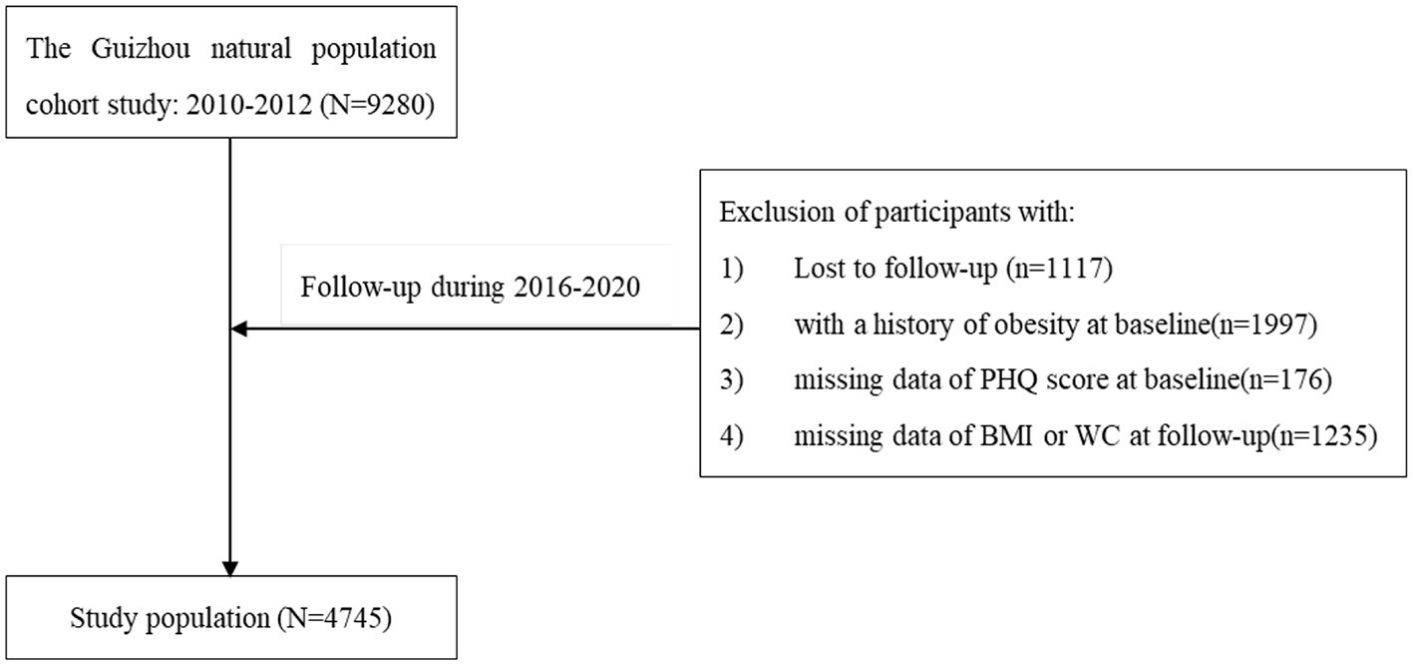
The flow chart.

The Guizhou Population Health Cohort Study (GPHCS) was a prospective community-based cohort conducted in Southwest China during 2010–2020 (18). Through the multistage proportional stratified cluster sampling method, 9,280 adult residents from 48 townships of 12 districts in Guizhou Province were recruited from November 2010 to December 2012. The inclusion criteria included: (1) Aged 18 and above; (2) Living in the study area and having no plans to move out; (3) Completing the questionnaire and blood sampling. All participants were subsequently followed up for major chronic diseases and vital status during 2016–2020, with a loss to follow-up rate of 12.04%. We further excluded 1,997 individuals with general and/or abdominal obesity at baseline (with BMI ≥ 28 kg/m^2^ or having a waist circumference of ≥85 cm for women, or ≥90 cm for men), 1,245 missing BMI or WC at follow-up, and 176 without sufficient information on depression at baseline. Finally, the remaining 4,745 participants were eligible for the analysis (Figure 1). This study was approved by the Institutional Review Board of Guizhou Province Centre for Disease Control and Prevention (No. s2017-02), and written informed consent was signed by all subjects. All deaths were confirmed through the death registration information system and the essential public health service system.

### 2.2. Data collection

Baseline information included sociodemographic characteristics (age, gender, ethnicity, education level, residence, marital status, and occupation), lifestyle (tobacco and alcohol consumption, physical activity), and chronic medical history (hypertension, dyslipidemia, diabetes mellitus, and cardiovascular diseases), which was collected by trained investigators through structured questionnaires *via* face-to-face interview.

Physical examination data, including height, weight, waist circumference, and blood pressure, were collected by trained investigators through standard procedures. Standing height was measured to the nearest 0.1 cm without shoes using a portable stadiometer. Weight was measured to the nearest 0.1 kg using a digital weighing scale. WC was measured to the nearest 0.1 cm at the midpoint between the lowest rib margin and the iliac crest. Blood pressure data were taken as the average value of three consecutive measurements. Venous blood samples were obtained in the early morning for fasting blood glucose, total cholesterol, high-density lipoprotein cholesterol, low-density lipoprotein cholesterol, and triglyceride levels after the participants had fasted for at least 8 h.

Above methods for data collection were same as baseline study during the follow-up study.

### 2.3. Assessments of depression and obesity

The Patient Health Questionnaire-9 (PHQ-9) was used to measure the levels of depression among participants according to the Diagnostic and Statistical Manual of Mental Disorders criteria (DSM-IV) (19). Subjects needed to answer nine questions which were graded from 0 to 3 and the total score ranged from 0 to 27, in which points = 0 was determined as non-depression, 1–4 points as minimal depression, and ≥5 points as mild or more advanced depression (20).

Body mass index (BMI) was calculated as weight in kg divided by height in m squared and general obesity was defined as BMI ≥ 28 kg/m^2^. Abdominal obesity was defined as a waist circumference of ≥85 cm for women and ≥90 cm for men (21). Obesity was defined if either of them was met. Overweight was defined based on BMI (24.0–27.9 kg/m^2^) or WC (80–85 cm for women and 85–90 cm for men).

### 2.4. Covariates

Alcohol consumption was defined as alcohol consumed at least once a month in the past 12 months. Physical activity was at least 150 min of moderate or high-intensity physical activity per week. Dietary habits were divided into two groups (light or greasy) according to whether participants consumed more than 5 g/day of salt or more than 25 g/ day of grease. Hypertension was defined as one of the following conditions: (1) systolic blood pressure (SBP) ≥ 140 mmHg and/or diastolic blood pressure (DBP) ≥90 mmHg; (2) Self-reported physician diagnosis of hypertension or having received antihypertensive treatment (22). Diabetes mellitus (DM) was defined as one of the following conditions: (1) Fasting blood glucose ≥ 7.0 mmol/l; (2) 2-h postprandial blood glucose ≥ 11.1 mmol/l; (3) Glycosylated hemoglobin ≥ 6.5%; (4) Self-reported physician diabetes diagnosis or receiving hypoglycemic treatment (23). Dyslipidemia was diagnosed as one of the following conditions: (1) Total cholesterol (TC) ≥ 6.22 mmol/l; (2) Triglycerides (TG) ≥ 2.26 mmol/l; (3) High-density lipoprotein cholesterol (HDL-C) <1.04 mmol/l; (4) Low-density lipoprotein cholesterol (LDL-C) ≥ 4.14 mmol/l; (5) Self-reported physician dyslipidemia diagnosis or having received lipid-lowering treatment (24).

### 2.5. Statistical analysis

Continuous variables were expressed in means and standard deviations, and categorical variables were as frequencies with proportions. χ^2^ test and Kruskal-Wallis test were used to compare the group differences of variables. Person-year of follow-up was calculated from the baseline survey to the date of confirmed death, obesity appears, or completion of follow-up, whichever came first. We fitted four Cox proportional hazards regression models to estimate hazard ratio (HR), the adjusted HR (aHR), and corresponding 95% confidence interval (CI) to determine the association between depression and the risk of obesity. Model 1: without any adjustment for covariates. Model 2: adjusted for age (<30, 30–59.9, ≥60 years) and gender (male or female). Model 3: model 2 added education (10 years and above), occupation (farmer or other), physical activity (yes or no), marriage (unmarried, married, divorced), family relations (Good, general, poor), alcohol use (yes or no), dietary habit (yes or no). Model 4: model 3 added baseline diabetes (yes or no), baseline hypertension (yes or no), and baseline dyslipidemia (yes or no). We tested for interactions between all target and adjustment variables and further performed stratified analysis if significant interactions were observed. Model 4 was repeated after individuals with overweight at baseline were excluded for sensitivity analyses. Schoenfeld residuals were used to test the hazard proportionality assumption in Cox regression models and no violation of proportionality was found. Two-sided *P* < 0.05 was considered statistically significant. All statistical procedures were performed in R software (Version 4.0.3; R Foundation for Statistical Computing, Vienna, Austria).

## 3. Results

### 3.1. Baseline characteristics

Among 4,745 adults in this study, 3,510 were not considered depressed, 922 were minimal depression, and 313 were mild or more advanced depression. Details of the baseline characteristics are shown in Table 1. The average age of all participants was 44.09 ± 15.07 years, and nearly half (48.8%) were male. More than half of them were Han Chinese (59.8%), farmers (54.4%), or had received education for ≥9 years (45.1%). Most (80.8%) were married. Differences between the non-depressed and those with different grades of depression were statistically significant (*p* < 0.05) in terms of age, gender, ethnicity, education time, occupation, and family relationship (seen in Table 1).

**Table 1:** General characteristics of Chinese adults without obesity at baseline over depression groups.

**Characteristics**	**Total** **(*N* = 4,745)**	**Depression**			***P*-value**

		**No (*****N*** = **3,510)**	**Minimal (*****N*** = **922)**	**Mild or more advanced (*****N*** = **313)**	
PHQ-9 score	0.88 ± 2.04	0	2.12 ± 1.06	7.12 ± 2.77	<0.001
Age, years	44.09 ± 15.07	43.38 ± 15.04	46.40 ± 15.19	45.20 ± 15.28	<0.001
<30	1,004 (21.2)	796 (22.7)	158 (17.1)	50 (16.0)	
30–59.9	2,995 (63.1)	2,192 (62.5)	588 (63.8)	215 (68.7)	
≥60	746 (15.7)	522 (14.8)	176 (19.1)	48 (15.3)	
Men, %	2,315 (48.8)	1,777 (50.6)	411 (44.6)	127 (40.6)	<0.001
Han Chinese, %	2,839 (59.8)	2,055 (58.5)	584 (63.3)	200 (63.9)	0.010
Education ≥ 9 years, %	2,141 (45.1)	1,654 (47.1)	368 (39.9)	119 (38.0)	<0.001
Farmer, %	2,583 (54.4)	1,999 (57.0)	462 (50.1)	122 (39.0)	<0.001
Alcohol use, %	1,537 (32.4)	1,085 (30.9)	346 (37.5)	106 (33.9)	<0.001
Greasy diet, %^*^	3,135 (66.3)	2,338 (66.8)	609 (66.3)	188 (60.5)	0.078
**Marriage, %**					0.023
Unmarried	488 (10.3)	390 (11.1)	69 (7.5)	29 (9.3)	
Married	3,835 (80.8)	2,815 (80.2)	769 (83.4)	251 (80.2)	
Divorced	422 (8.9)	305 (8.7)	84 (9.1)	33 (10.5)	
Physical activity, %^*^	288 (6.1)	228 (6.5)	45 (4.9)	15 (4.8)	0.114
**Family relation, %**					<0.001
Good	3,908 (82.4)	3,012 (85.8)	716 (77.7)	180 (57.5)	
General	766 (16.1)	457 (13.0)	189 (20.5)	120 (38.3)	
Poor	71 (1.5)	41 (1.2)	17 (1.8)	13 (4.2)	
Hypertension, %	1,122 (23.6)	806 (23.0)	241 (26.1)	75 (24.0)	0.129
Diabetes, %^*^	358 (7.6)	258 (7.4)	73 (8.0)	27 (8.7)	0.620
Dyslipidemia, %	2,541 (53.6)	1,848 (52.6)	522 (56.6)	171 (54.6)	0.092
BMI, kg/m^2^	22.10 ± 2.44	22.11 ± 2.44	22.10 ± 2.45	21.92 ± 2.39	0.402
WC, cm	74.31 ± 7.13	74.38 ± 7.15	74.26 ± 7.20	73.66 ± 6.68	0.220

^*^Missing value exists.

PHQ-9, Patient Health Questionnaire-9; BMI, body mass index; WC, waist circumference.

### 3.2. Associations between baseline depression and incident obesity

Totally, 4,745 subjects were followed up for 34,138.66 person-years, with an average follow-up of 7.19 ± 1.15 person-years and a maximum of 9.54 person-years. One thousand one hundred fifteen incident obesity were identified with an incidence of 32.66 per 1,000 PYs, 1,063 (31.14 per 1,000 PYs) and 321 (9.40 per 1,000 PYs) for abdominal obesity and general obesity, respectively. The incidence of obesity was highest in subjects with mild or more advanced depression (38.01 per 1,000 PYs). As shown in Table 2, both model 1 (univariate cox model) and model 2 (adjusted for age and gender) showed that depression was associated with an increased risk of incident obesity. In the fully adjusted models, the aHR was 1.07 for abdominal obesity with per SD increase of PHQ score. Compared with those not depressed (PHQ score = 0), participants with minimal (aHR: 1.22, 95% CI: 1.05, 1.43) and mild or more advanced depression (aHR: 1.27, 95% CI: 1.01, 1.62) remained at higher risks of incident abdominal obesity. Risks of any incident obesity among subjects with minimal (aHR: 1.21, 95% CI: 1.04, 1.40), mild or more advanced depression (aHR: 1.30, 95% CI: 1.03, 1.64) were also significantly higher than those among not depressed participants (seen in Table 2).

**Table 2:** Associations between depression at baseline and incident obesity among Chinese adults.

**Obesity**	**PHQ score**	**Cases, *n***	**Incident rate/1,000 PYs**	**aHR (95% CI)**			

				**Model 1**	**Model 2**	**Model 3**	**Model 4**
Abdominal obesity (WC)	PHQ-score (per SD increase)			1.09 (1.03, 1.15)^**^	1.06 (1.01, 1.12)^*^	1.07 (1.01, 1.14)^*^	1.07 (1.01, 1.14)^*^
	No (0)	754	29.77	1.00	1.00	1.00	1.00
	Minimal (1-4)	229	34.97	1.29 (1.12, 1.50)^***^	1.22 (1.05, 1.42)^**^	1.21 (1.04, 1.41)^*^	1.22 (1.05, 1.43)^**^
	Mild or more advanced (≥5)	80	35.36	1.30 (1.03, 1.64)^*^	1.19 (0.94, 1.50)	1.28 (1.01, 1.63)^*^	1.27 (1.01, 1.62)^*^
General obesity (BMI)	PHQ-score (per SD increase)			1.06 (0.95, 1.18)	1.05 (0.94, 1.16)	1.03 (0.93, 1.15)	1.03 (0.93, 1.15)
	No (0)	240	9.48	1.00	1.00	1.00	1.00
	Minimal (1-4)	55	8.40	1.01 (0.75, 1.35)	0.98 (0.73, 1.31)	0.93 (0.69, 1.25)	0.93 (0.69, 1.25)
	Mild or more advanced (≥5)	26	11.49	1.41 (0.94, 2.12)	1.34 (0.89, 2.01)	1.34 (0.87, 2.04)	1.33 (0.87, 2.03)
Obesity (WC or BMI)	PHQ-score (per SD increase)			1.09 (1.03, 1.15)^***^	1.06 (1.01, 1.12)^*^	1.07 (1.01, 1.13)^*^	1.07 (1.01, 1.13)^*^
	No (0)	792	31.27	1.00	1.00	1.00	1.00
	Minimal (1-4)	237	36.19	1.28 (1.10, 1.48)^***^	1.21 (1.04, 1.40)^*^	1.20 (1.03, 1.39)^*^	1.21 (1.04, 1.40)^*^
	Mild or more advanced (≥5)	86	38.01	1.34 (1.07, 1.67)^*^	1.23 (0.98, 1.54)	1.31 (1.04, 1.65)^*^	1.30 (1.03, 1.64)^*^

Model 1: Without any adjustment for covariates.

Model 2: Adjusted for age (<30, 30–59.9, ≥60 years), gender.

Model 3: Model 2 plus education, occupation, physical activity, marriage, family relations, alcohol use, dietary habit.

Model 4: Model 3 plus hypertension, diabetes mellitus, dyslipidemia.

^***^P < 0.001, ^**^P < 0.01, ^*^P < 0.05.

PHQ-9, Patient Health Questionnaire-9; PY, person years; HR, hazard ratio; BMI, body mass index; WC, waist circumference; 95% CI, 95% confidence interval; SD, standard deviation.

### 3.3. Stratification analysis

**Figure 2:**
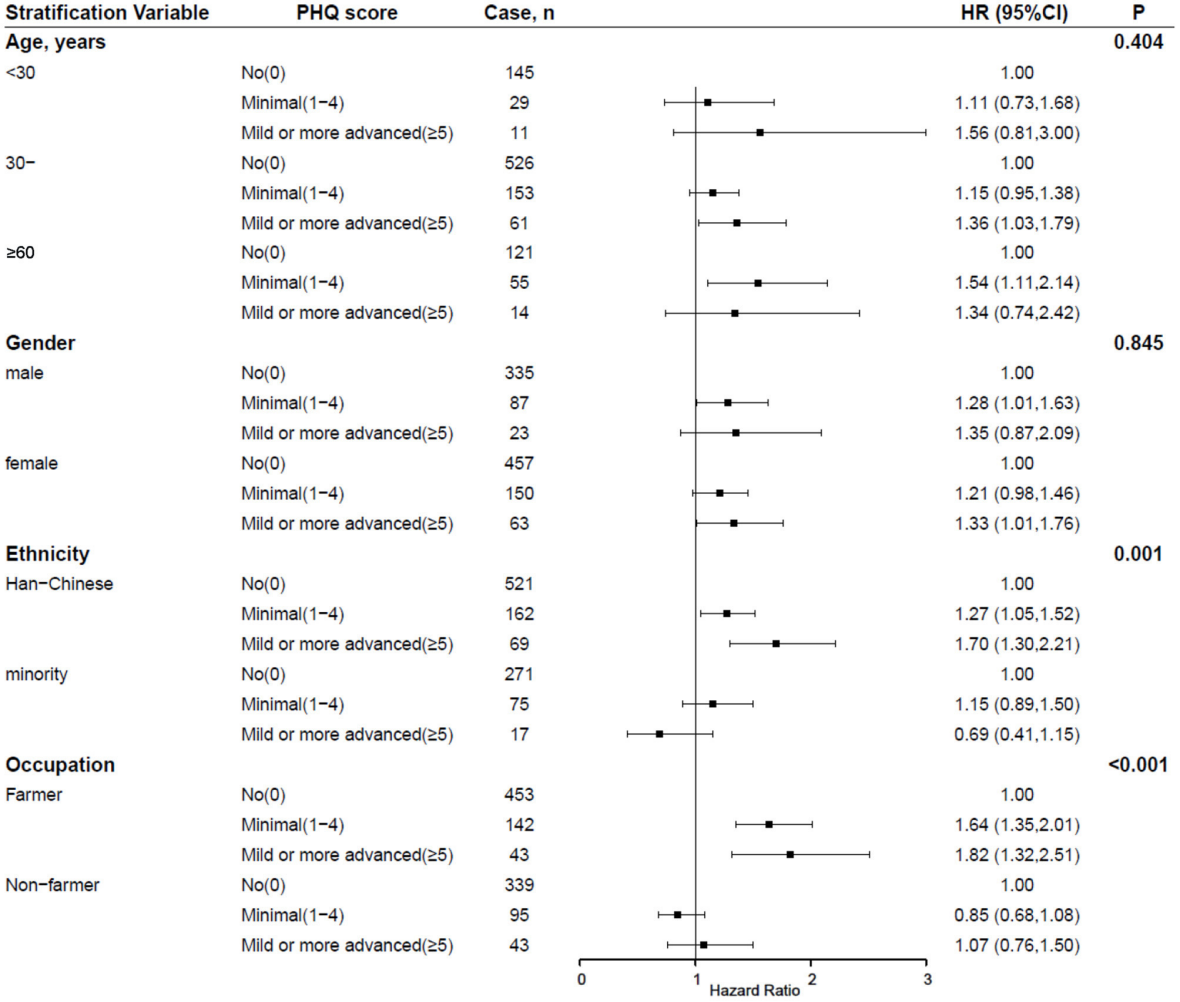
Interactions between depression and sociodemographic factors on the incident obesity among Chinese adults. Adjusted for age, gender, education, occupation, physical activity, marriage, family relations, alcohol use, dietary habit, hypertension, diabetes mellitus, and dyslipidemia. PHQ-9, Patient Health Questionnaire-9; aHR, adjusted hazard ratio; 95% CI, 95% confidence interval.

The potential modification effects of age, gender, ethnicity, and occupation on the association of depression with incident obesity were explored in this study (seen in Figure 2). Associations between depression and incident obesity significantly varied over ethnicity and occupation (*P* for interaction = 0.001 and <0.001, respectively), and risks for incident obesity were statistically higher in Han Chinese or farmers. However, age and gender interactions were not observed.

**Figure 3:**
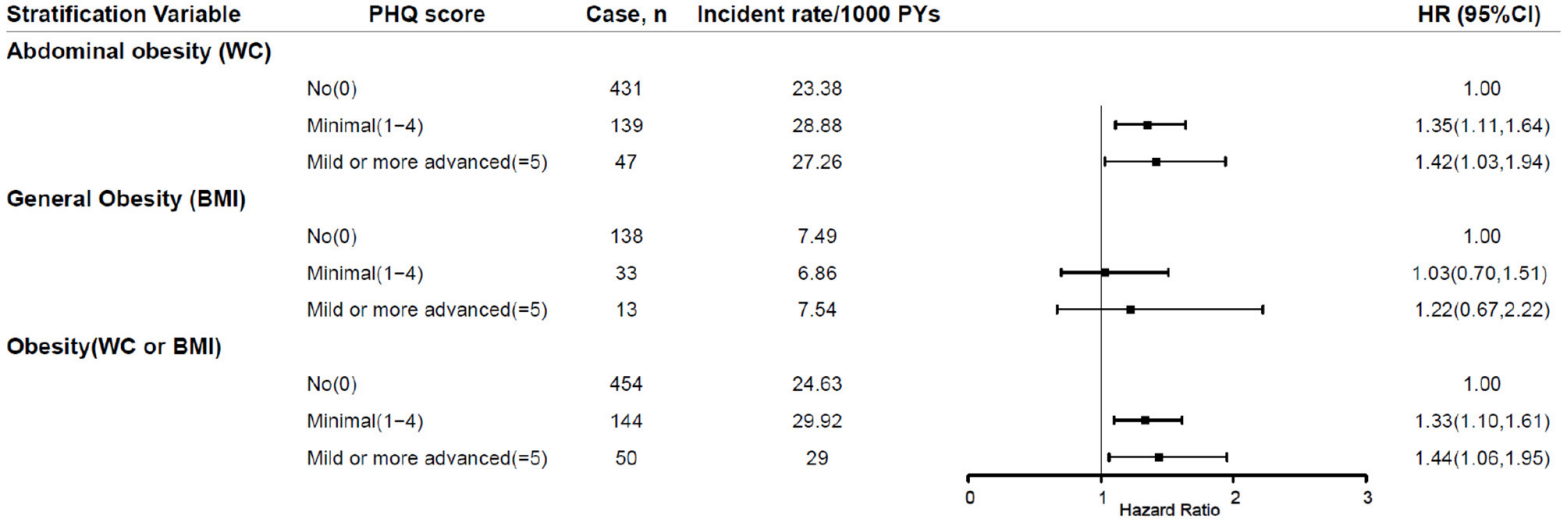
Sensitivity analyses after exclusion of individuals with overweight at baseline. PHQ-9, Patient Health Questionnaire-9; PY, person years; HR, hazard ratio; BMI, body mass index; WC, waist circumference; 95% CI, 95% confidence interval.

In the sensitivity analysis, the corresponding effect estimates of baseline depression status on the incident obesity did not change substantially after excluding participants with overweight at baseline (seen in Figure 3).

## 4. Discussion

Based on a prospective cohort study in Southwest China, we found that the incidence rate of incident obesity was high and depression was strongly associated with the risk of incident obesity among this community adult population, especially among Han Chinese and farmers. Our findings indicated that improving depression may help to prevent and control developing obesity.

The incidence rate of abdominal obesity (31.1/1,000 PYs) was higher than general obesity (9.4/1,000 PYs) in this study. Compared with another earlier cohort study whose incidence rate of general obesity was 6.97‰ in China (25), that was lower than our findings and may be driven by economic developments, sociocultural norms and policies with China's rapid urbanization and industrialization (26). Previous studies and WHO data have shown that the prevalence of obesity in many countries doubled and even quadrupled over the last 30 years (1, 27). The critical increase of obesity in China and worldwide called that more vigorous interventions should be implemented for obesity prevention and treatment.

Previous studies have demonstrated a positive association of depression and obesity (10, 28, 29). Apart from that, the limitation of BMI measures has been proven as unable to discriminate between fat percent and lean mass (30), so weight management guidelines in several countries suggested health professionals consider both BMI and WC to diagnose obesity (31). WC has confirmed exist higher accuracy in the measurement of depression-induced obesity compared with BMI due to the accumulation of visceral adipose (29). In this study, aHR of incident abdominal obesity was a 1.07 per SD (2.04 score) increase in PHQ score, which was comparable with that from two cohort studies in Norway and USA (32, 33). Several potential mechanisms on the association between depression and obesity were studied. Apart from the effect on individual perception of weight (34), impaired fat metabolism caused by abnormal secretion of the hypothalamic-pituitary-adrenal axis (32), and intake of antidepressants such as tricyclic and selective serotonin reuptake inhibitors may explain the mechanism of obesity (35, 36). Hyperphagia and hypersomnia have been proved to be critical features of atypical depression (37), which lead to weight gain through increased energy intake and circadian rhythm dysregulation (38). A twin study in Washington demonstrated that shared genetic risk might act upon depression and obesity (39), which also should be considered. However, this study did not observe a significant association between depression and incident general obesity.

Several researches indicated that the association between being obese and depressed mood tended to vary across ethnic groups (40), which suggested that the sociocultural differences or ethnicity moderation effects should be considered (36). The increased risk of incident obesity based on BMI and WC was more significant among Han Chinese and farmers in this study, where such ethnic variations may be explained by genetic background and other factors (40, 41). Miao and Bouyei were the main of the 1,906 minorities accounted for 40.2% in this study and their genetic background had been reported to exhibit significant differences compared with Han Chinese (42). One study conducted in Guizhou province also pointed that Bouyei people had a lower prevalence of general (4.8 vs. 10.9%) and abdominal obesity (13.6 vs. 26.8%) compared with Han Chinese (43), which was similar to this study. The more significant association among farmers may be related to the low awareness of obesity and depression, and inadequate access to depression-related healthcare services while other occupational groups could be improved by timely management of depression-related symptoms (44). A French study showed that the prevalence of depression combined with obesity was higher in rural areas (45).

The presence of effect modification by gender has been reported for the association between depression and obesity, which was not observed in this study. A cohort study in Houston showed that depressed males had a 6-fold increased risk of obesity while another meta-analysis demonstrated that the association was more pronounced in adolescent females (28, 46). Accumulating evidence tends to hold a stronger correlation between depression and obesity among females (11). The divergences of psychological characteristics, interpersonal barriers, and physical predispositions might explain the association among females (39, 47, 48). Physiological studies indicated that the difference was a consequence of the combined action of stronger immune responses and more inflammatory markers caused by the increment of estrogen (49). Intense mood swings and emotional eating also act as mediators between depression and future weight gain, which were more common among the female (11, 48). However, no gender interaction was observed in this study.

To our knowledge, this was the first study to investigate the association between depression and incident obesity among the Chinese community population in southwest China. Strengths of this study were the prospective cohort design with the 10-year follow-up period and the relatively low loss to follow-up rate. Also, we explored the effects of depression on the risk of incident obesity with anthropometric indices through standardized measurements rather than self-report. Of course, this study had some notable limitations. First, baseline depression was assessed by the PHQ-9 scale without clinical diagnoses. Second, data on depression status and antidepressant use were not collected during the follow-up survey, both of which may bias the findings of this study. Third, some possible confounding factors such as the family history, genetic variants of obesity and energy intake were not collected and controlled well in this study, which should be considered in future studies. Our findings in this southwest Chinese population need to be confirmed by more prospective or intervention studies over different populations. Further studies on clinically diagnosed depression and repeated measures of depression are required to confirm the complex bidirectional association between depression and obesity among Chinese population.

## 5. Conclusions

In conclusion, the long-term prospective study demonstrated that there were high risks in the incident obesity among the Chinese community population in southwest China, and both minimal and mild or more advanced depression increased the risk of developing obesity, especially in Han Chinese and farmers. Our findings further suggest that improving healthcare for depression may benefit to prevent and control the developing obesity, especially for abdominal obesity, in the community settings. Government departments and medical institutions, especially community health service centers should pay more attention to farmers' mental health issues, and developing appropriate community-based depression intervention services to improve their obesity control.

## Data availability statement

The raw data supporting the conclusions of this article will be made available by the authors, without undue reservation.

## Ethics statement

The studies involving human participants were reviewed and approved by Institutional Review Board of (or Ethics Committee) Guizhou Province Centre for Disease Control and Prevention (No. S2017-02). The patients/participants provided their written informed consent to participate in this study. Written informed consent was obtained from the individual(s) for the publication of any potentially identifiable images or data included in this article.

## Author contributions

TL and BW: conceptualization, methodology, formal analysis, validation, writing—original draft, and visualization. YC: methodology, data curation, writing—review, and editing. YY and JZ: conceptualization, methodology, supervision, funding acquisition, writing—review, and editing. NW and KX: conceptualization, methodology, data curation, writing—review, and editing. CF: conceptualization, methodology, supervision, resources, writing—review, and editing. All authors contributed to manuscript revision, read, and approved the submitted version.
